# An electrochemical biosensor for the detection of epithelial-mesenchymal transition

**DOI:** 10.1038/s41467-019-14037-w

**Published:** 2020-01-10

**Authors:** Xin Du, Zhenhua Zhang, Xiaodi Zheng, Hongyan Zhang, Dan Dong, Zhenguo Zhang, Min Liu, Jun Zhou

**Affiliations:** 1 Shandong Provincial Key Laboratory of Animal Resistance Biology, Collaborative Innovation Center of Cell Biology in Universities of Shandong, Institute of Biomedical Sciences, College of Life Sciences, Shandong Normal University, Jinan, Shandong 250014 China; 2 State Key Laboratory of Medicinal Chemical Biology, College of Life Sciences, Nankai University, Tianjin, 300071 China

**Keywords:** Sensors and biosensors, Biosensors

## Abstract

Epithelial-mesenchymal transition (EMT) is critically involved in a variety of biological processes. Electrochemical sensing offers potential to develop more effective technology for EMT detection. In this study, by using the unique performance of quantum dot (QD)-nanocomposite materials, we establish an electrochemical biosensor that can specifically detect the change of E-cadherin and analyze different stages of EMT. The signal for EMT is largely magnified due to the transmission of molecular information to the electronic device. In addition, differential pulse voltammetry reveals that the response of the electrochemical signals is rapid and sensitive, due to the synergistic effect of QDs and carbon nanotube-gold nanoparticles. Our study thus suggests that electrochemical sensing is an effective technology for detecting EMT and may have broad applications in analyzing various cell type transitions.

## Abstract (web summary)

Epithelial-mesenchymal transition (EMT) plays a key role in embryonic development, wound healing and cancer. Here the authors develop an electrochemical sensor to detect EMT using E-cadherin antibody-quantum dot conjugates and a carbon nanotube-gold nanoparticle-modified electrode as a detection platform.

## Introduction

Epithelial–mesenchymal transition (EMT) plays an important role in diverse biological processes, such as embryonic development, tissue morphogenesis, and wound healing^1,2^. In addition, there is accumulating evidence demonstrating a critical role for EMT in tumor progression; EMT facilitates the infiltration of tumor cells to surrounding tissues and metastasis to distant sites^2–4^. During EMT, epithelial cells lose polarity and intercellular junctions, present with slender morphology, and acquire cell motility^5,6^. A number of molecules, such as transforming growth factor β (TGF-β) and epidermal growth factor, have been identified as EMT triggers^7,8^. In addition, several molecular events have been implicated in the EMT process, including the activation of transcription factors, expression of specific proteins, and rearrangement of the cytoskeleton^9–11^.

Downregulation of E-cadherin, a member of the cadherin family, is one of the key events in EMT initiation^10,12^. E-cadherin expression has been demonstrated to negatively correlate with the pathological classification and staging of lung, liver, ovarian, and gastric cancers and, therefore, has been used as a biomarker for tumor diagnosis^13–15^. CdSe/ZnS quantum dots (QDs) are excellent tools to prepare an electrochemical biosensor for EMT, because of its unique characteristics of high-electron density and size-dependent, tunable and narrow fluorescence emission spectra, which allow the synchronous detection of fluorescent and electrochemical signals in cells^16,17^.

In this study, we develop an electrochemical biosensor of EMT, by using E-cadherin antibody-QD (E-cad-Ab-QD) conjugates as dual optical/electrochemical labels and a carbon nanotube-gold nanoparticle (CNT-AuNP)-modified electrode as the detection platform. Our electrochemical sensing system can detect the change of E-cadherin specifically, rapidly, and sensitively. In addition, this biosensor can examine cells at different EMT stages and distinguish circulating tumor cells and cells from tumor tissues in situ. The biosensor can also analyze the EMT process for cells in different fluids. Our results suggest that electrochemical sensing is a convenient and sensitive technique for the detection of EMT.

## Results

### Establishment of the EMT model system

**Fig. 1:**
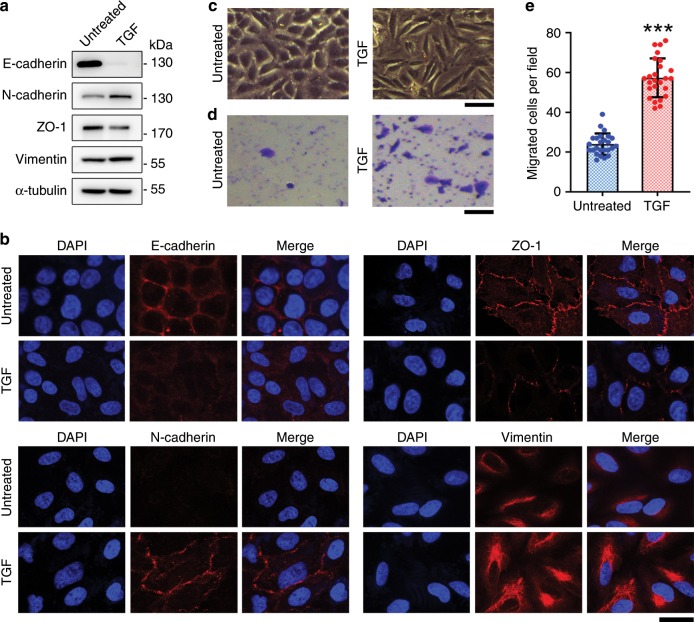
Establishment of the EMT model. **a** A549 cells treated or untreated with TGF were analyzed by immunoblotting with E-cadherin, N-cadherin, ZO-1, vimentin, and α-tubulin antibodies. **b** Examination of E-cadherin, N-cadherin, ZO-1, and vimentin by immunofluorescence microscopy. Scale bar, 20 μm. **c** Bright field images of A549 cells treated or untreated with TGF. Scale bar, 100 μm. **d** Transwell migration assays with A549 cells treated or untreated with TGF. Scale bar, 100 μm. **e** Analysis of the number of migrated cells in the visual field. ***p < 0.001 by Student’s *t* test. Error bars represent ± s.d. (*n* = 25). Source data are provided as a Source Data file.

Given that EMT is well studied in lung cancer, we used A549 human lung cancer cells in this study. We analyzed various markers of EMT following treatment of cells with TGF-β1 (hereinafter referred to as TGF), to characterize the establishment of a cellular EMT model system using electrochemical detection. Immunoblot analysis revealed downregulation of E-cadherin by TGF treatment, whereas N-cadherin was upregulated (Fig. 1a), illustrating the classical cadherin switch during EMT^14,18^. Downregulation of ZO-1 and upregulation of vimentin were also in accordance with changes in classical EMT markers (Fig. 1a). In agreement with the immunoblotting results, immunofluorescence staining showed expected changes in the levels of EMT markers, including E-cadherin, N-cadherin, ZO-1, and vimentin (Fig. 1b).

We also compared cell morphology images before and after TGF treatment in a bright field. We observed that upon TGF treatment, the shape of A549 cells changed from epithelial pebbles to elongated mesenchymal spindles (Fig. 1c), which could facilitate cell migration and is a defining behavioral property of cells that have undergone EMT. Transwell migration assays revealed that the number of cells migrating across the membrane was significantly higher in the TGF-treated group than the untreated group (Fig. 1d, e). These results indicate the successful establishment of the EMT model system.

### Electrochemical performance of the modified electrode

**Fig. 2:**
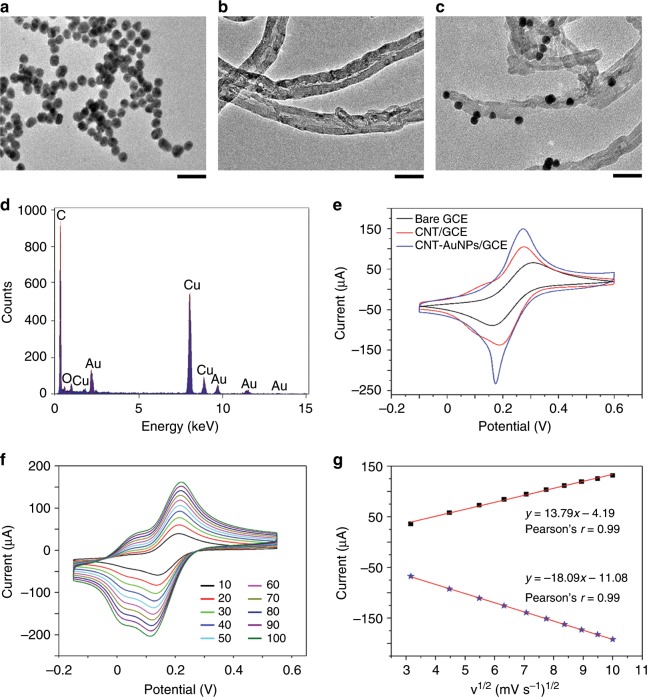
Characterization of the electrochemical performance of the modified electrode. **a**–**c** Transmission electron microscopy images of AuNPs **a**, CNT **b**, and CNT-AuNPs **c**. Scale bar, 50 nm. **d** X-ray diffraction profiles of CNT-AuNPs. **e** Cyclic voltammetry curves of bare GCE, CNT/GCE, and CNT-AuNPs/GCE recorded in 10 mm K_3_[Fe(CN)_6_] at a scan rate of 50 mV s^−1^. **f** Kinetic analysis of CNT-AuNPs/GCE at scan rates ranging from 10 to 100 mV s^−1^ indicating that reaction of the modified electrode is a diffusion-controlled surface reaction. **g** Linear fits of the oxidized peak current (Ipa) and reduced peak current (Ipc) versus the square root of the scan rate (v^1/2^).

Aiming to enhance the electrode performance, we used a multi-walled carbon nanotube-gold nanoparticle (MWCNT-AuNP) nanocomposite to modify the surface. The morphology and structure of MWCNT-AuNPs were characterized using transmission electron microscopy. Pure AuNPs were demonstrated as homogeneous spherical structures, with an average diameter of 5 nm (Fig. 2a). The typical core-shell structure of MWCNTs without aggregation was clearly displayed with the help of poly(diallyldimethylammonium chloride), which is a strong cationic polyelectrolyte (Fig. 2b). Owing to electrostatic adsorption, AuNPs with a negative charge attached to the surface of CNTs (Fig. 2c). Energy-dispersive X-ray spectroscopy was performed to analyze the elemental composition of the nanomaterial, showing the obvious peaks of Au and C corresponding to AuNP and CNT, respectively; the peaks associated with Cu and O originated from the substrate (Fig. 2d).

The electrochemical performance of bare glassy carbon electrode (GCE), CNT/GCE, and CNT-AuNPs/GCE was investigated using cyclic voltammetry, which demonstrated an apparent redox peak ascribed to a ferricyanide ion for each modified electrode (Fig. 2e). We calculated the microscopic electroactive area of CNT-AuNPs/GCE according to the Randles–Sevcik equation^19^, which was 2.6- and 1.7-times higher than that of bare GCE and CNT/GCE, respectively. These results illustrate that the modified nanomaterial can enhance the conductivity and sensibility of the electrode.

The kinetics of the modified electrode was investigated by analyzing the effects of the scan rate on the redox current. The electrochemical performance of CNT-AuNPs/GCE was examined in a 10 mm potassium ferricyanide solution, with scan rates ranging from 10 to 100 mV s^−1^. Maximal current values of the redox reaction increased linearly with increasing scan rate. In addition, the distance between redox peaks became farther and farther apart (Fig. 2f). Based on these results, we performed a linear fit about the oxidation peak (Ipa) and reduction (Ipc) peak currents related to the square root of the scan rate (v^1/2^). The ultimate linear equations were determined to be Ipa = 13.79v^1/2^–4.19 and Ipc = −18.09v^1/2^–11.08 (Fig. 2g). The results from these calculations demonstrate that the electrochemical signal was the result of a diffusion-controlled surface reaction^20^.

### Specificity of the E-cad-Ab-QD probe

**Fig. 3:**
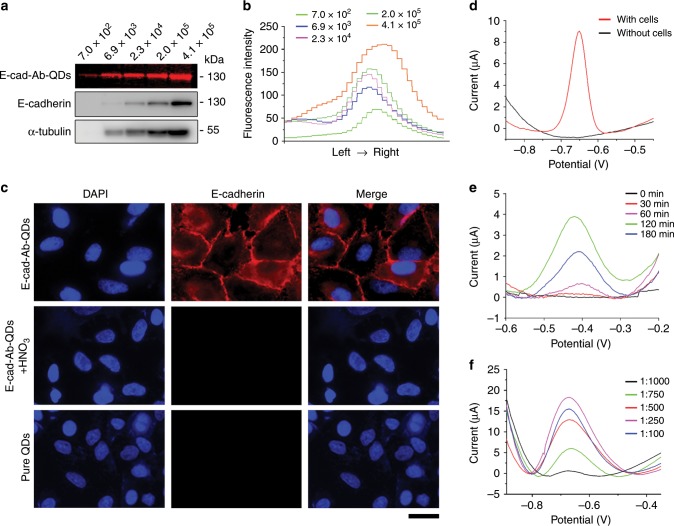
Specificity and sensitivity of the prepared probe. **a** Immunoblotting of E-cadherin with the E-cad-Ab-QD probe and standard E-cadherin antibodies using different amount of A549 cells. **b** Fluorescence intensity curves of E-cadherin from different amount of cells. **c** Immunofluorescence microscopy of A549 cells stained with the E-cad-Ab-QD probe, the E-cad-Ab-QD probe with HNO_3_ solution, and pure QDs. Scale bar, 25 μm. **d** Response currents of the electrochemical biosensor measured with or without A549 cells by DPV. **e** Optimization of the incubation time for the E-cad-QD probe. **f** Optimization of the E-cad-Ab-QD probe concentration for detecting E-cadherin. Source data are provided as a Source Data file.

To explore the validity of this probe, we first considered whether E-cad-Ab-QD could specifically recognize E-cadherin protein, which is a prerequisite for EMT detection. We compared E-cadherin obtained from different amount of cells using E-cad-Ab-QDs and standard E-cadherin antibodies. The polyvinylidene difluoride membranes stained with E-cad-Ab-QDs were brighter than those stained with standard E-cadherin antibodies (Fig. 3a), and the fluorescence intensity increased with increasing amount of cells (Fig. 3b). Immunofluorescence microscopy also demonstrated that the prepared probe could specifically bind to E-cadherin and that the fluorescence signal disappeared after QDs were dissolved by HNO_3_ (Fig. 3c). Moreover, cells incubated with pure QDs lacked a fluorescence signal (Fig. 3c). Electrochemical detection was also performed with and without A549 cells, showing that the specific electrochemical signal to the probe at − 0.65 V was related to E-cadherin on the cell surface (Fig. 3d).

We then studied the effects of incubation time on the E-cad-Ab-QD probe, which could directly affect the performance of electrochemical biosensors. Differential pulse voltammetry (DPV) experiments using the electrochemical EMT system prepared with different incubation time showed that the oxidation currents increased from 0 to 120 min and then decreased over time (Fig. 3e). Thus, an incubation time of 120 min was selected for the probe. The effect of probe concentration on the performance of the biosensor was also assessed with the dilution ratio varying from 1:1000 to 1:100. We found that the oxidation currents increased from 1:1000 dilution to 1:250 dilution and then decreased (Fig. 3f). Based on these results, we selected 1:250 as the optimal dilution ratio of the E-cad-Ab-QD probe for the detection of EMT.

### Analytical performance of the EMT-sensing system

**Fig. 4:**
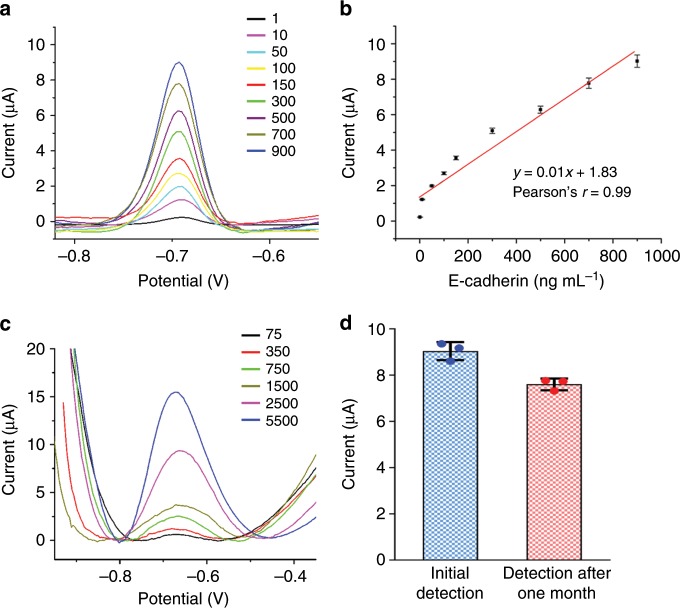
Detection of E-cadherin at different protein levels and with different amount of cells. **a** DPV detection of varying concentrations (1–900 ng mL^−1^) of purified E-cadherin protein. **b** Calibration curve showing the linear relationship of the biosensor in detection of E-cadherin. **c** DPV detection of varying amount of A549 cells. **d** Measurement of the stability of the biosensor for detecting E-cadherin. Current responses were obtained using the initial E-cad-QD probe and the probe stored for 1 month. Error bars represent ± s.d. (*n* = 3). Source data are provided as a Source Data file.

The analytical performance of the proposed EMT-sensing system was first evaluated at the protein level. DPV measurements showed that the peak current signal of QDs was aggrandized with increasing concentrations of purified E-cadherin protein (Fig. 4a). This increase in current is attributed to more adsorption of the E-cad-Ab-QD probe by the E-cadherin antigen. The proposed biosensor exhibited clear linearity with E-cadherin concentrations from 1 to 900 ng mL^−1^, and the linear regression equation was calculated as *y* = 0.01*x* + 1.83 (Fig. 4b). To prove that detection of the oxidation peak is a valid means to assess the level of E-cadherin in cells, different amount of A549 cells was examined using the biosensor. When the amount of cells gradually increased from 75 to 5500, the current peak significantly increased (Fig. 4c), suggesting that the limit of detection of the sensor is 75 cells. These results indicate that the proposed sensing system can be used to detect E-cadherin and analyze the EMT process. The stability of the prepared biosensor for the detection of E-cadherin was evaluated using the E-cad-Ab-QD probe stored for 1 month, and 83% of the initial current response was retained (Fig. 4d), suggesting that the sensor has exceptional stability.

### Detection of different EMT stages

**Fig. 5:**
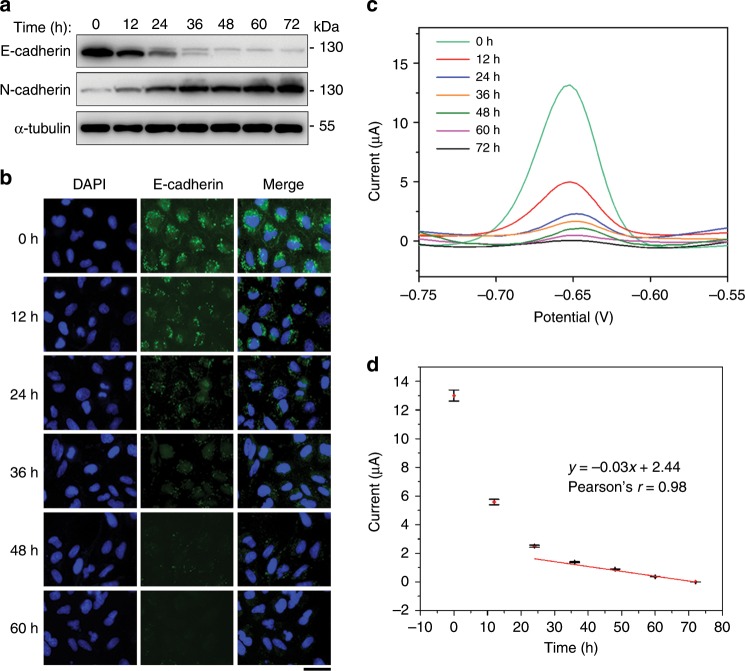
Detection of different EMT stages with the electrochemical biosensor. **a** Immunoblotting of E-cadherin, N-cadherin, and α-tubulin in A549 cells treated with TGF for different time. **b** Examination of E-cadherin intensity in A549 cells upon TGF treatment by immunofluorescence microscopy. Scale bar, 40 μm. **c** Electrochemical detection of different EMT stages using the biosensor by DPV. **d** Calibration curve showing the relationship between current and incubating time. Error bars represent ± s.d. (*n* = 3). Source data are provided as a Source Data file.

To further investigate the electrochemical performance of the EMT biosensor, different EMT stages were established by treatment of A549 cells with TGF for different time and detected with immunoblotting, immunofluorescence microscopy, and the electrochemical method. Immunoblotting showed that E-cadherin and N-cadherin levels changed as expected, demonstrating the EMT process (Fig. 5a). Immunofluorescence microscopy showed gradual reduction of E-cadherin fluorescence intensity with increasing induction time (Fig. 5b). DPV measurements showed that the peak current signal became smaller at later stages of EMT (Fig. 5c). We fitted the calibration curve of current and induction time after five independent repetitive experiments, which gave a linear current response at late EMT stages (Fig. 5d). The current signal displayed a sharp decline in early EMT stages (Fig. 5d), in agreement with the immunoblotting results (Fig. 5a). These data demonstrate that the electrochemical sensor could distinguish different stages of EMT.

### Practicability of the EMT-sensing system

**Fig. 6:**
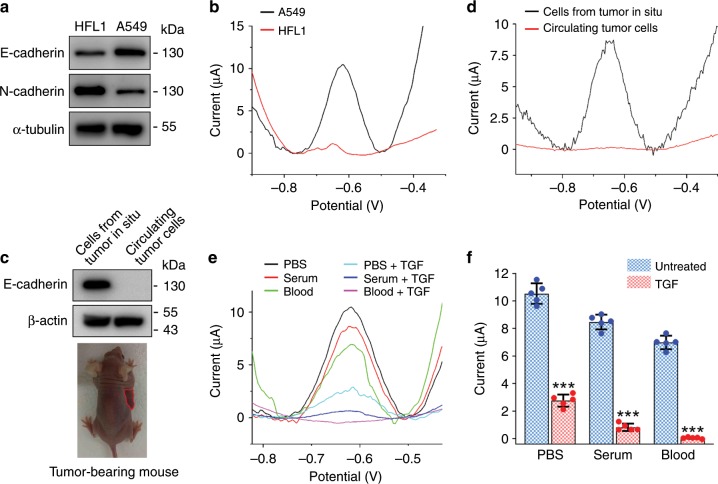
Examination of the practicability of the EMT biosensor. **a** Immunoblot analysis of HFL1 and A549 cells with E-cadherin, N-cadherin, and α-tubulin antibodies. **b** Electrochemical detection of HFL1 and A549 cells with the EMT biosensor. **c**, **d** Immunoblot analysis of E-cadherin and β-actin **c** and DPV measurement **d** using cells from tumor in situ and circulating tumor cells. **e** Electrochemical detection of E-cadherin using A549 cells in PBS, fetal calf serum, and mouse blood. **f** Experiments were performed as in **e**, and the peak currents were then analyzed. ***p < 0.001 by Student’s *t* test. Error bars represent ± s.d. (*n* = 5). Source data are provided as a Source Data file.

To evaluate the practicability of the EMT-sensing system, we compared its applications in the detection of A549 cells and HFL1 human lung fibroblasts. Immunoblot analysis of E-cadherin and N-cadherin levels confirmed the epithelial feature of A549 cells and mesenchymal feature of HFL1 cells (Fig. 6a). Consistent with the immunoblotting results, DPV measurements with the EMT biosensor revealed a remarkable difference in the currents of A549 and HFL1 cells (Fig. 6b). Next, we sought to use the biosensor to examine cells prepared from tumor tissues inoculated in situ and circulating tumor cells. Immunoblotting showed that the level of E-cadherin was high in cells from tumor tissues in situ, but was not detectable in circulating tumor cells (Fig. 6c). This observation was confirmed by DPV experiments with the EMT biosensor (Fig. 6d). We then used the biosensor to analyze A549 cells in different fluids, including phosphate-buffered saline (PBS), serum, and blood. We found that the biosensor could detect TGF-induced changes of currents for cells in these three different fluids, although the currents were reduced for cells in serum and blood, compared with those in PBS, most likely owing to the interfering substances in serum and blood (Fig. 6e, f).

### Detection of EMT using different cell lines

**Fig. 7:**
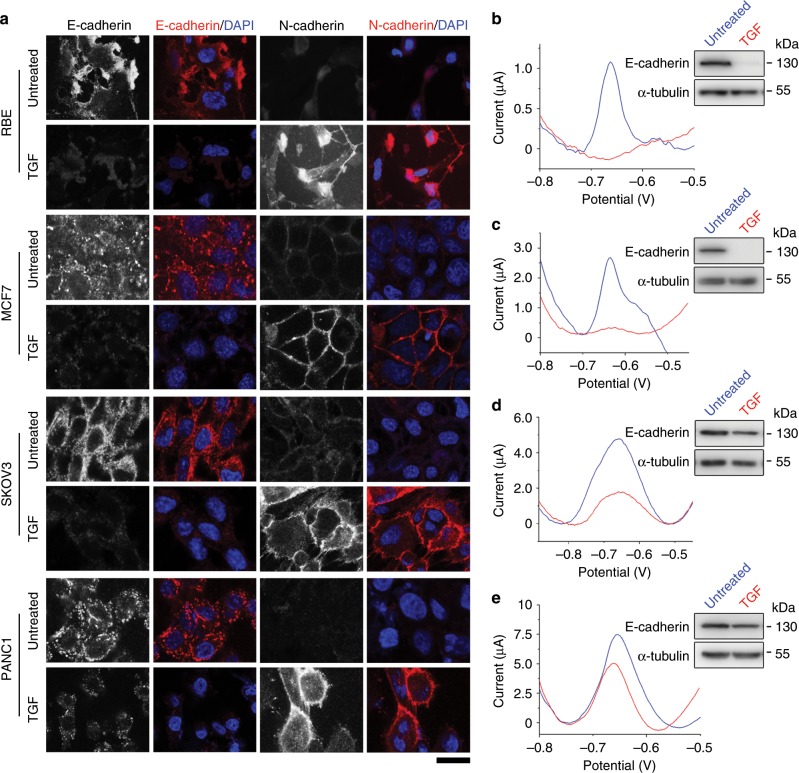
The EMT biosensor is applicable in multiple cell lines. **a** Immunofluorescence microscopy of E-cadherin and N-cadherin in RBE, MCF7, SKOV3, and PANC1 cells treated or untreated with TGF. Scale bar, 25 μm. **b**–**e** Electrochemical detection of RBE **b**, MCF7 **c**, SKOV3 **d**, and PANC1 **e** cells using the prepared biosensor. Upper right, immunoblots showing E-cadherin and α-tubulin levels in cells treated or untreated with TGF. Source data are provided as a Source Data file.

To verify the universal application of the prepared EMT electrochemical sensor, we analyzed E-cadherin levels in RBE liver cancer, MCF7 breast cancer, SKOV3 ovarian cancer, and PANC1 pancreatic cancer cell lines before and after TGF treatment. Immunofluorescence microscopy revealed that TGF treatment significantly decreased E-cadherin levels and increased N-cadherin levels in all the four cell lines, suggesting that these cells underwent EMT (Fig. 7a). In agreement with the immunofluorescence microscopy results, immunoblotting showed the reduction of E-cadherin in these cell lines upon TGF treatment (Fig. 7b–d). In addition, DPV measurements showed that the electrochemical signals in RBE (Fig. 7b), MCF7 (Fig. 7c), SKOV3 (Fig. 7d), and PANC1 (Fig. 7e) cells were obviously weakened after TGF treatment. These results demonstrate that the EMT electrochemical sensor can be used for EMT detection in multiple cell lines.

## Discussion

EMT is a cellular process critical for embryogenesis, organogenesis, and cancer development^21,22^. EMT is mainly detected using traditional methods, such as immunoblotting and immunofluorescence microscopy^23^. However, these methods have several disadvantages, including long detection time, cumbersome steps, and high cost. Thus, it is important to develop a convenient and rapid method to detect EMT.

In this study, we have designed an electrochemical biosensor capable of detecting EMT using E-cadherin as a biomarker. QD and CNT-AuNP nanocomposites were employed to enhance the sensitivity of the EMT biosensor^24,25^. The prepared electrochemical biosensor has several advantages over conventional methods. First, the probe containing E-cadherin antibodies in the EMT biosensor can specifically recognize E-cadherin in living cells; therefore, the biosensor could distinguish different stages of EMT. Second, simultaneous fluorescence detection could be carried out while detecting electrochemical signals because of the unique performance of QDs. The electrochemical and fluorescent signals of the probe are more sensitive than traditional methods. Finally, the electrochemical detection of EMT has no need for cell lysis, fixation, and incubation with secondary antibodies, thereby saving time and cost.

In summary, we have developed an electrochemical biosensor that allows the detection of EMT, in a shorter amount of time and with higher sensitivity in living cells compared with standard methods. In addition to the analysis of EMT, the biosensor system can be extended to examine a variety of cellular processes, such as cell differentiation, senescence, and death.

## Methods

### Chemicals and reagents

CNTs (30–50 nm diameter, 10–20 μm average length) were purchased from Xfnano Materials Tech (Nanjing, China). Qdot 655 ITK carboxyl QDs (Q21321MP) were obtained from Thermo Fisher Scientific (Waltham, MA, USA). Boric acid, potassium chloride (KCl), and phosphate were all obtained from Sinopharm Group Chemical Reagent (Shanghai, China). E-cadherin (ab1416), N-cadherin (ab18203), and α-tubulin (ab18251) antibodies were supplied by Abcam (Cambridge, MA, USA). ZO-1 (66452-1-Ig) and vimentin (10366-1-AP) antibodies were from Proteintech (Rosemont, IL, USA), and β-actin antibodies (AB2001) were from Abways (Shanghai, China). All primary antibodies were used at a dilution of 1:2000 for immunoblotting and 1:1000 for immunofluorescence microscopy. Horseradish peroxidase-conjugated anti-rabbit (AB0101, Abways) and anti-mouse (115-035-003, Jackson ImmunoResearch, West Grove, PA, USA) secondary antibodies were used at a dilution of 1:5000 for immunoblotting. Rhodamine-conjugated anti-rabbit (111-025-003), rhodamine-conjugated anti-mouse (115-025-003), and fluorescein-conjugated anti-mouse (115-095-003) secondary antibodies were from Jackson ImmunoResearch and used at a dilution of 1:1000 for immunofluorescence microscopy. Purified E-cadherin protein (5085) and chloroauric acid (HAuCl_4_) were purchased from Sigma-Aldrich (St. Louis, MO, USA). Fetal calf serum was purchased from Biological Industries (Cromwell, CT, USA). Blood was collected from the tail vein of mice. Double distilled water was applied to prepare all detection systems.

### Cell culture and construction of the EMT model

A549 (TCHu150), PANC1 (TCHu98), MCF7 (TCHu74), RBE (TCHu179), SKOV3 (TCHu185), and HFL1 (GNHu28) cell lines were purchased from the cell bank of the Chinese Academy of Sciences (Shanghai, China). All cell lines were authenticated and checked regularly for preservation of their typical morphology and behavior. A549, PANC1, and MCF7 cells were cultured in Dulbecco's Modified Eagle medium supplemented with 10% fetal bovine serum (FBS). RBE and SKOV3 cells were cultured in RPMI1640 medium supplemented with 10% FBS. HFL1 cells were cultured in F12K medium supplemented with 10% FBS. Cells were grown in a humidified atmosphere at 5% CO_2_ and 37 °C. Cells were seeded in six-well plates for immunoblotting and 24-well plates for immunofluorescence staining and electrochemical detection. EMT was induced by addition of TGF (100-21, PeproTech, London, UK) to the medium at a final concentration of 15 ng mL^−1^ and incubation for 48 h. Tumor cells were inoculated subcutaneously into the armpit of right anterior limb of female BALB/c mice, and circulating tumor cells were obtained by capture with a vein indwelling needle^26^. Mouse experiment protocols were approved by the Institutional Animal Care and Use Committee of Shandong Normal University.

### Immunofluorescence microscopy

Cells grown on chamber slides incubated with or without TGF were washed twice with PBS and then fixed with 4% paraformaldehyde for 20 min. After permeabilization with 0.2% Triton X-100/PBS and blocking with 4% BSA for 1 h at room temperature, cells were incubated and stained with antibodies overnight. After washing with PBS, slides were incubated with secondary antibodies for 2 h at room temperature. Cell nuclei were visualized with 4,6-diamidino-2-phenylindole (Sigma-Aldrich). Staining of living cells was performed by incubation with E-cad-Ab-QDs for 2 h prior to the fixation step.

### Immunoblot analysis

Whole-cell extracts were resolved separately on sodium dodecyl sulphate-polyacrylamide gel electrophoresis gels and transferred onto polyvinylidene difluoride membranes. Membranes were blocked with 5% skim milk powder for 2 h, followed by incubation with primary antibodies in 5% skim milk powder at 4 °C overnight. After removing excess primary antibodies by washing the membranes in Tris-buffered saline containing 0.1% Tween-20 (TBST, 3 × 5 min each), secondary antibodies conjugated with horseradish peroxidase were incubated with the membranes for 1 h at room temperature. Membranes were then washed in TBST and visualized with enhanced chemiluminescence detection reagent (Thermo Fisher Scientific). Immunoblot analysis using E-cad-Ab-QDs was performed without secondary antibodies and was excited by blue light.

### Transwell migration assays

Cells (1 × 10^5^ cells per well) starved for 24 h were placed in a transwell chamber with 500 µL medium containing 10% FBS in the lower layer of a 24-well plate. After incubation at 37 °C for 24 h, cells that migrated across the membrane were fixed by methanol, stained with 0.1% crystal violet, and quantified under a microscope.

### Fabrication of the E-cad-Ab-QD probe

E-cad-Ab-QD nanocomposites were prepared, with modifications, according to QD product specification. In brief, the stock solution (10 μL) of carboxyl quantum dots were diluted to 2 mL using 10 mm borate buffer (pH 7.4). A total of 30 µL E-cad-Ab was added to the above solution followed by immediate addition of 1.2 μL of 5 mg mL^−1^ 1-ethyl-3-[3-dimethylaminopropyl]carbodiimide hydrochloride stock solution. The solution was gently stirred for 1–2 h at room temperature for conjugation. The conjugate solution was filtered through a 0.22 μm polyethersulfone syringe filter to remove large aggregates and washed at least five times using 50 mm borate buffer (pH 8.3). The solution was concentrated to 20 µL and stored at 4 °C.

### Electrode modification

The surface of the GCE was polished by alumina powders with diameters of 0.3 and 0.05 μm to remove oxide layers. To remove other physically adsorbed substances, the electrode surface was ultrasonically washed using double distilled water and ethanol. The GCE was immediately dried under nitrogen gas. CNTs were ultrasound-dispersed in 0.4% poly-diallyldimethylammonium chloride for 1 h and then centrifuged and washed using double distilled water at least three times. The GCE surface was treated by placing 10 μL of the CNT suspension (3 mg mL^−1^) in a 20 mL solution (0.1 mm l-cysteine, 0.1 m H_2_SO_4_, 5 mm HAuCl_4_) followed by chronoamperometry at 400 mV for 120 s to prepare CNT-AuNPs.

### Electrochemical measurements

**Fig. 8:**
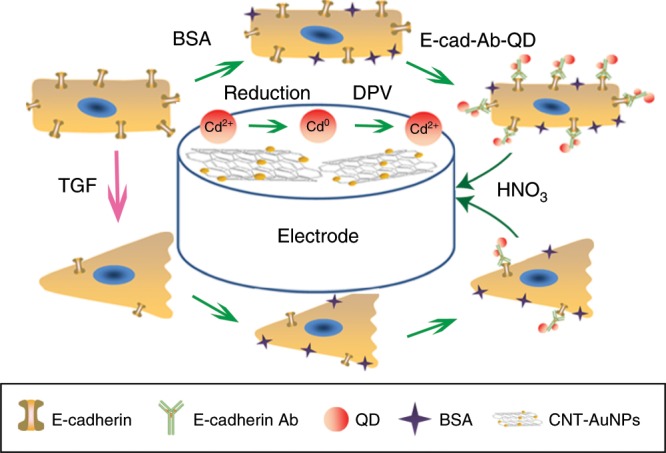
Construction of the electrochemical biosensor of EMT.

Electrochemical experiments were performed with a CHI400C Potentiostat electrochemical workstation (CH Instruments, Chenhua, Shanghai, China). A CNT-AuNP-modified GCE served as the working electrode, Ag/AgCl (saturated KCl) as the reference electrode, and platinum wire (1-mm diameter) as the counter electrode. Cyclic voltammetry detection was performed between − 0.1 V and 0.6 V at 50 mV s^−1^ in potassium ferricyanide solution (10 mm). Cells were incubated with the E-cad-Ab-QD probe for 2 h and then washed three times with PBS. QDs were dissolved with 0.1 m HNO_3_. Following a preconcentration step at − 1.1 V for 300 s, the DPV detection of QDs was conducted from − 1.0 V ~ 0 V in a Hg^2+^-acetic acid buffer (pH 5.2) solution with a frequency of 15 Hz, amplitude of 25 mV, and potential step of 4 mV. The electrochemical detection process is illustrated in Fig. 8. All electrochemical experiments were performed at room temperature.

### Statistical analyses

Student’s *t* test was performed for comparison between different groups, and all data were presented as the mean ± s.d. of at least three independent experiments.

### Reporting summary

Further information on research design is available in the Nature Research Reporting Summary linked to this article.

## Supplementary information

Reporting Summary

## Data Availability

All data that support the findings of this study are available from the corresponding authors upon reasonable request. The source data underlying Figs. 1a, e, 3a, 4b, d, 5a, d, 6a, c, f, and 7b–e are provided as a Source Data file.

## Source data

Source Data
