# Supplementary material for: An electrochemical biosensor for the detection of epithelial-mesenchymal transition
Source: Nat Commun. 2020 Jan 10;11:192. doi: 10.1038/s41467-019-14037-w (PMC6954246; doi:10.1038/s41467-019-14037-w)
Supplement: Supplementary file 1 — Reporting Summary [file 41467_2019_14037_MOESM1_ESM.pdf]

nature research

Corresponding author(s): Min Liu and Jun zhou

Last updated by author(s): Dec 9, 2019

Reporting Summary

Nature Research wishes to improve the reproducibility of the work that we publish. This form provides structure for consistency and transparency in reporting. For further information on Nature Research policies, see Authors & Referees and the Editorial Policy Checklist .

Statistics

For all statistical analyses, confirm that the following items are present in the figure legend, table legend, main text, or Methods section.

n/a	Confirmed	
	x	The exact sample size (n) for each experimental group/condition, given as a discrete number and unit of measurement
	x	A statement on whether measurements were taken from distinct samples or whether the same sample was measured repeatedly
	x	The statistical test(s) used AND whether they are one- or two-sided<br>*Only common tests should be described solely by name; describe more complex techniques in the Methods section.*
x		A description of all covariates tested
x		A description of any assumptions or corrections, such as tests of normality and adjustment for multiple comparisons
	x	A full description of the statistical parameters including central tendency (e.g. means) or other basic estimates (e.g. regression coefficient) AND variation (e.g. standard deviation) or associated estimates of uncertainty (e.g. confidence intervals)
x		For null hypothesis testing, the test statistic (e.g. F, t, r) with confidence intervals, effect sizes, degrees of freedom and P value noted<br>*Give P values as exact values whenever suitable.*
x		For Bayesian analysis, information on the choice of priors and Markov chain Monte Carlo settings
x		For hierarchical and complex designs, identification of the appropriate level for tests and full reporting of outcomes
	x	Estimates of effect sizes (e.g. Cohen's d, Pearson's r), indicating how they were calculated

Our web collection on statistics for biologists contains articles on many of the points above.

Software and code

Policy information about availability of computer code

Data collection	The data in the study was collected using CHI400C Potentiostat electrochemical workstation, Leica SP8 confocal microscope, and GE Healthcare Amersham Imager 600.
Data analysis	The data in the study was analyzed using the the softwares of OriginPro 2016 and Photoshop.

For manuscripts utilizing custom algorithms or software that are central to the research but not yet described in published literature, software must be made available to editors/reviewers. We strongly encourage code deposition in a community repository (e.g. GitHub). See the Nature Research guidelines for submitting code & software for further information.

Data

Policy information about availability of data

All manuscripts must include a data availability statement. This statement should provide the following information, where applicable:

- Accession codes, unique identifiers, or web links for publicly available datasets
- A list of figures that have associated raw data
- A description of any restrictions on data availability

All data that support the findings of this study are available from the corresponding authors upon reasonable request. The source data underlying Figs 1a, 1e, 3a, 4b, 4d, 5a, 5d, 6a, 6c, 6f and 7b-e are provided as a Source Data file.

Field-specific reporting

Please select the one below that is the best fit for your research. If you are not sure, read the appropriate sections before making your selection.

x

Life sciences study design

All studies must disclose on these points even when the disclosure is negative.

Sample size	We examined varying concentrations of cells ranging from 7.0 ×10^2 to 4.1×10^5 cells.
Data exclusions	No data were excluded from the analysis in the study.
Replication	We have successfully repeated the experiment for at least three times.
Randomization	The sample of every type cell detected in our study was random drawn in cell suspension.
Blinding	Yes.

Reporting for specific materials, systems and methods

We require information from authors about some types of materials, experimental systems and methods used in many studies. Here, indicate whether each material, system or method listed is relevant to your study. If you are not sure if a list item applies to your research, read the appropriate section before selecting a response.

Materials & experimental systems

n/a	Involved in the study	
☐	☒	Antibodies
☐	☒	Eukaryotic cell lines
☒	☐	Palaeontology
☒	☐	Animals and other organisms
☒	☐	Human research participants
☒	☐	Clinical data

Methods

n/a	Involved in the study	
☒	☐	ChIP-seq
☒	☐	Flow cytometry
☒	☐	MRI-based neuroimaging

Antibodies

Antibodies used	E-cadherin (ab1416), N-cadherin (ab18203), and α-tubulin (ab18251) antibodies were supplied by Abcam (Cambridge, MA, USA). ZO-1 (66452-1-Ig) and vimentin (10366-1-AP) antibodies were from Proteintech (Rosemont, IL, USA), and β-actin antibodies (AB2001) were from Abways (Shanghai, China). All primary antibodies were used at a dilution of 1:2000 for immunoblotting and 1:1000 for immunofluorescence microscopy. Horseradish peroxidase-conjugated anti-rabbit (AB0101, Abways) and anti-mouse (115-035-003, Jackson ImmunoResearch, West Grove, PA, USA) secondary antibodies were used at a dilution of 1:5000 for immunoblotting. Rhodamine-conjugated anti-rabbit (111-025-003), rhodamine-conjugated anti-mouse (115-025-003), and fluorescein-conjugated anti-mouse (115-095-003) secondary antibodies were from Jackson ImmunoResearch and used at a dilution of 1:1000 for immunofluorescence microscopy.
Validation	The antibodies used in this study were validated.

Eukaryotic cell lines

Policy information about cell lines

Cell line source(s)	A549 (TCHu150), PANC1 (TCHu98), MCF7 (TCHu74), RBE (TCHu179), SKOV3 (TCHu185), and HFL1 (GNHu28) cell lines were purchased from the cell bank of the Chinese Academy of Sciences (Shanghai, China).
Authentication	All cell lines were authenticated and checked regularly for preservation of their typical morphology and behavior.
Mycoplasma contamination	All cell lines used in this study were negative for mycoplasma contamination.
Commonly misidentified lines (See ICLAC register)	No.
